# Acceptability of a Digital Care App in Patients Undergoing Hip and Knee Arthroplasty: Prospective Cohort Study

**DOI:** 10.2196/79682

**Published:** 2026-01-27

**Authors:** Yacine Louni, Matthew Laroche, Abdulrhman Alnasser, Mohammad Abuhaneya, Eric Belzile, Sandhya Baskaran, Jennifer Mutch, Anthony Albers

**Affiliations:** 1 Faculty of Medicine, McGill University, Montreal, QC, Canada; 2 Division of Orthopaedic Surgery, McGill University, St Mary’s Hospital, 3830 Avenue Lacombe, Montreal, QC, H3T 0A2, Canada, +1 514-345-3511 ext 3282; 3 St Mary's Hospital Research Center, St Mary's Hospital, Montreal, QC, Canada

**Keywords:** mobile health, orthopedics, patient satisfaction, telemedicine, telerehabilitation, total joint arthroplasty

## Abstract

**Background:**

Mobile health (mHealth) apps have become more commonly used in orthopedics. However, for these apps to be efficient, patients should be willing to use them, making it essential to understand patients’ perspectives of mHealth interventions.

**Objective:**

The aim of this single-center, intent-to-treat, preoperative single-cohort study of 100 patients was to evaluate the acceptability of mymobility (Zimmer-Biomet), an mHealth app designed for the postoperative care of total hip arthroplasty (THA) and total knee arthroplasty (TKA).

**Methods:**

We measured acceptability using the theoretical framework for acceptability (TFA) preoperatively and at 3 months post operation. We also measured satisfaction with app use postoperatively using the Usefulness, Satisfaction, and Ease of Use questionnaire as well as patient-reported outcome measures preoperatively and postoperatively using the Oxford hip and knee scores and the visual analog scale for pain. Patients included were 18 years or older; underwent unilateral primary total hip, total knee, or partial knee arthroplasty; spoke and read French or English; and had a smartphone with internet access. Participants used mymobility in addition to standard government-funded physiotherapy.

**Results:**

The preoperative overall TFA result was 4.2 out of 5, but results decreased significantly postoperatively. There was higher self-efficacy in preoperative TFAs with higher education, and lower acceptability in postoperative TFAs with TKA. The Usefulness, Satisfaction, and Ease of Use questionnaire revealed a good level of satisfaction with the use of the app. Patient-reported outcome measures showed earlier improvement in THA (31.2 d) than in TKA (89.4 d), whereas the visual analog scale showed a rapid decrease in pain with both procedures. Only 1 patient expressed privacy concerns with the use of the app.

**Conclusions:**

There was a good level of acceptability with the use of mymobility for the postoperative management of THA and TKA, although acceptability decreased postoperatively. This decrease could signify high expectations toward the app preoperatively or higher than expected difficulty and pain in the early postoperative period. Acceptability tended to increase with higher education and decrease with TKA. These trends are consistent with prior literature and constitute a potential gap to address for app developers. The influence of the natural recovery process on acceptability remains unclear. Future studies could explore this gap by comparing results in cohorts using the app to cohorts with standard care.

## Introduction

In Canada, there were 60 705 hip and 70 379 knee replacements performed in the inpatient setting in 2023‐2024 [1]. These procedures were the third and second most common inpatient surgeries, and these numbers are expected to at least double in the United States (and likely Canada) over the next 15 years due to an aging population [2,3]. Another growing sector in health care is the use of smartphones for patient care. An estimated 71% of the world population uses a smartphone in 2024, and there are more than 350 000 mobile health (mHealth) apps currently available [4,5]. This increasing use of mHealth apps is also present in various areas of orthopedics, where numerous studies have shown that they are efficient and convenient, that they promote patient adherence and engagement with their treatment, and that they facilitate rehabilitation while promoting positive outcomes [6-13].

Although mHealth has the potential to improve outcomes, patients’ attitudes toward these apps must be assessed, as they can only be effective if patients are willing to use them. User acceptability is defined as “a multifaceted construct that reflects the extent to which people delivering or receiving health care intervention consider it to be appropriate based on anticipated or experienced cognitive and emotional responses to the intervention” [14]. Acceptability is an important measure, as it not only provides information on patients’ experience with mHealth apps, but also gives an insight into their cognitive and emotional response [14]. This helps determine whether patients genuinely perceive them as appropriate and valuable.

Recent studies investigated patients’ perspectives of mHealth interventions in the context of postoperative hip and knee arthroplasty care. These studies found a high level of engagement and increased compliance with treatment exercises when using mHealth interventions [13,15-18]. Other findings include participants being satisfied with these interventions while also finding them easy to use and engaging, and providing better connection to their treating team [13,16,19,20]. These results are promising and could signify that patients are willing to use mHealth apps for their rehabilitation. However, these studies either did not assess acceptability or did so through surveys that were researcher-created or not primarily designed to measure acceptability. According to Perski et al [21], this reduces clarity regarding which aspects of acceptability are satisfied, and which are not. Additionally, high engagement does not necessarily reflect patients’ perceptions of the intervention. Engagement might be driven by external factors, such as study participation, financial incentives, or free access to the devices and apps, while patients might still find the app to be time-consuming, repetitive, and burdensome.

Measuring acceptability, especially preoperatively, is therefore relevant as it provides a better perspective of patients’ opinions prior to and beyond use, and because it “may usefully be considered an emergent property […] which in turn influences (and is influenced by) user engagement and intervention effectiveness” [21].

We started using the mymobility app (Zimmer Biomet, Warsaw, and Indiana) in 2022 in our community hospital to provide postoperative care to patients receiving outpatient total hip arthroplasty (THA) and total knee arthroplasty (TKA) [22]. This app, available on iOS and Android, currently stands at more than 50 000 downloads on the Google Play Store and is amongst the most downloaded when compared to other apps with the same objective [23]. In this context, our team sought to investigate the acceptability of mymobility, an mHealth app specifically designed for the postoperative care of THA and TKA, using a standardized framework.

## Methods

### Study Design

The primary aim of this single-center, intent-to-treat (ITT), single-cohort study was to evaluate pre- and postoperative patient acceptability with the use of the mymobility app for the postoperative care of THA and TKA. The secondary outcomes were postoperative patient satisfaction with the app and pre- and postoperative patient-reported outcome measures (PROMs).

### Data Collection

Data collected for this study are presented in Multimedia Appendix 1. Demographic data were obtained from patient charts. PROMs were collected either directly through the mymobility app or by one of the authors contacting participants by phone or email. Details of the specific PROMs are provided later in this section. All data were stored securely and deidentified prior to analysis.

### Ethical Considerations

This study received ethics approval from the Research Ethics Board of the Montreal West Island’s Integrated University Health and Social Services Centers (Biomedical Subcommittee), affiliated with St. Mary’s Hospital, Montreal (IRB# 2024-954). All participants provided informed consent prior to participation. Study data, including personal information, remained confidential and were deidentified. No secondary analyses were conducted. Study participants received no financial compensation, but access to the mymobility app was given for free. No identifiable images of participants were included in this manuscript. Under the Personal Information Protection and Electronic Documents ACT, the data collected by mymobility could not be used or shared with third parties, nor could cookies be collected, unless participants gave their explicit consent within the app [24,25]. If consent was given, data could be used or shared by the app following anonymization, with consent revocable at any time. Participants were not required to give consent to data use or sharing, or to cookie collection to use the app and be part of the study. This was made clear to participants as part of their informed consent.

### App Description

Mymobility is an app that is accessible either through a smartphone or an Apple Watch. The app features educational content on pre- and postoperative care, exercise routines with metrics to track progress, AI-assisted gait speed analysis to determine the level of recovery, in-app PROMs, and telemedicine with the opportunity to send SMS text messages, videos, or pictures to health care providers (see Multimedia Appendix 2). The app engages patients in their recovery by sending notifications and reminders if users accept them while also keeping track of progress made. It also provides patients with basic and important smartphone functions to use (eg, Wi-Fi) for those with low digital literacy.

### Questionnaires

#### Acceptability

Acceptability was measured using the theoretical framework for acceptability (TFA) questionnaire developed by Sekhon et al [26], which was adapted to our research question (see Multimedia Appendix 3). The questionnaire comprises 8 items answered by participants preoperatively and at 3 months post operation by phone or email. Each statement was evaluated using a 5-point Likert scale: scores 1 to 2 were classified as negative, 3 as neutral, and 4‐5 as positive. The mean score was calculated for each of the 8 items, as well as an overall score.

#### Satisfaction

Patient satisfaction was measured using the Usefulness, Satisfaction, and Ease of Use (USE) questionnaire at 3 months post operation (see Multimedia Appendix 4) [27]. Satisfaction was defined as “the net feeling of pleasure or displeasure that results from aggregating all the benefits that a person hopes to receive from interaction with the information system” [27]. The questionnaire was answered by phone or email. Statements were evaluated using a 7-point Likert scale: scores 1 to 3 were classified as negative, 4 as neutral, and 5 to 7 as positive. The mean score was then calculated for each of the 4 sections of the questionnaire. Two additional questions assessing satisfaction were asked and scored on a 5-point Likert scale (see Multimedia Appendix 5).

#### Patient-Reported Outcome Measures

PROMs were measured using the Oxford Hip Score (OHS) and the Oxford Knee Score (OKS) as well as the visual analog scale (VAS) for pain (scored from 0 to 10) preoperatively and at 1, 3, and 6 months post operation (see Multimedia Appendices 6 and 7) [28-30]. OHS and OKS were answered directly within the app, whereas the VAS was answered by phone or email.

### Recruitment

Patients were included in the study if they were booked for unilateral primary THA or TKA, were clinically suited for telehealth care (characterized as age ≥18 y, speak and read French or English, and have a smartphone or a tablet), had internet access, and provided informed consent to participate in the study. Patients were excluded if they did not meet the inclusion criteria, if they were unable or unwilling to provide informed consent, if they were undergoing emergency THA or TKA, or if they were undergoing revision surgeries. Participants had access to the app in addition to normal postoperative care, which included physiotherapy and postoperative follow-up visits with an orthopedic surgeon.

Patients were approached by a member of the research team during their preoperative assessment visit in the clinic. Eligible patients were provided with an informed consent form detailing the study. Once enrolled, a 1-year free access to the mymobility app was provided to the participants by the treating physician or a member of the research team, after which an email with instructions to download the app was sent. Participants were then required to download the app and agree to the terms and conditions to use it [24].

In the end, 100 participants were included in this study and had a profile created in the app. Participants who did not download the app preoperatively were sent a reminder email and given a few days following surgery, after which they were considered as nondownloaders. In the end, 75 participants downloaded the app and 25 did not. Only participants who downloaded the app were provided with the study questionnaires, as the goal was to assess acceptability, satisfaction, and PROMs with use of the app. Patients who did not download the app remained in the study in the context of the ITT format. However, since deciding not to download the app could signify low acceptability, we contacted participants who did not download the app by phone or email to ask the reason behind their decision.

### Data Analysis

Study data was analyzed using the ITT principle. Data analysis was conducted and reported using STATA 17.0 and R software. Statistical significance was defined as a *P* value <.05.

## Results

**Figure 1.**
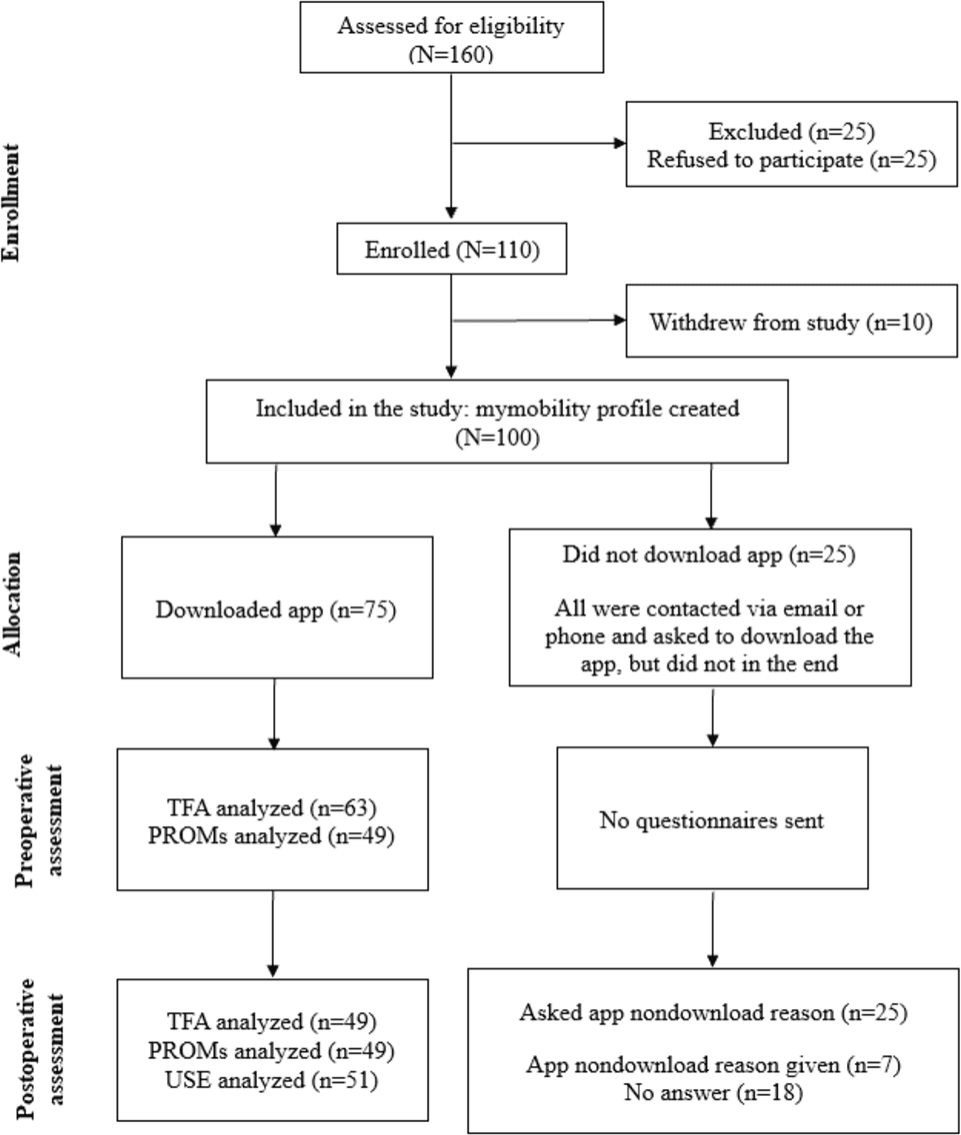
Study enrollment, participation, and questionnaire distribution flow diagram. PROM: patient-reported outcome measure; TFA: theoretical framework for acceptability; USE: Usefulness, Satisfaction, and Ease of Use.

The recruitment process is summarized in Figure 1. Baseline characteristics of our population can be found in Table 1.

**Table 1.** Baseline demographic and clinical characteristics of the study population.

Baseline variables	Participants (N=100)
Female, n (%)	51 (52)
Missing	1 (1)
Age (years), mean (SD)	67.0 (10.7)
Ethnicity, n (%)	
Caucasian	75 (82)
Asian	9 (10)
Arab	3 (3)
Other	5 (5)
Missing	8 (8)
Education, n (%)	
Graduate	20 (21)
Undergraduate	27 (29)
Postsecondary (nonuniversity)	25 (27)
Primary/secondary	22 (23)
Missing	6 (6)
Employment, n (%)	
Full/part time	31 (34)
Retired	56 (61)
Unemployed	5 (5)
Missing	8 (8)
Language used, n (%)	
English	64 (69)
French	23 (25)
Both	6 (6)
Missing	7 (7)
Comorbidities, n (%)	
Yes^a^	39 (39)
None	61 (61)
Hypertension	18 (31)
Diabetes	10 (17)
Obesity	6 (10)
Thyroid	4 (7)
Cholesterol	4 (7)
Other	17 (29)
Procedure, n (%)	
TKA^b^	55 (55)
THA^c^	42 (42)
PKA^d^/UKA^e^	3 (3)
Income (CAD $^f^), n (%)	
<25,000	8 (9)
25,000‐50,000	15 (17)
50,000‐75,000	10 (11)
>100,000	24 (27)
Prefer not to say/unknown	13 (15)
Missing	11 (11)
Area of residency, n (%)	
Urban	72 (77)
Rural	22 (23)
Missing	6 (6)

a Participants who answered “Yes” can have multiple comorbidities.

b TKA: total knee arthroplasty.

c THA: total hip arthroplasty.

d PKA: partial knee arthroplasty.

e UKA: unicompartmental knee arthroplasty.

f A currency exchange rate of CAD $1=US $0.75 is applicable.

### Acceptability

**Figure 2.**
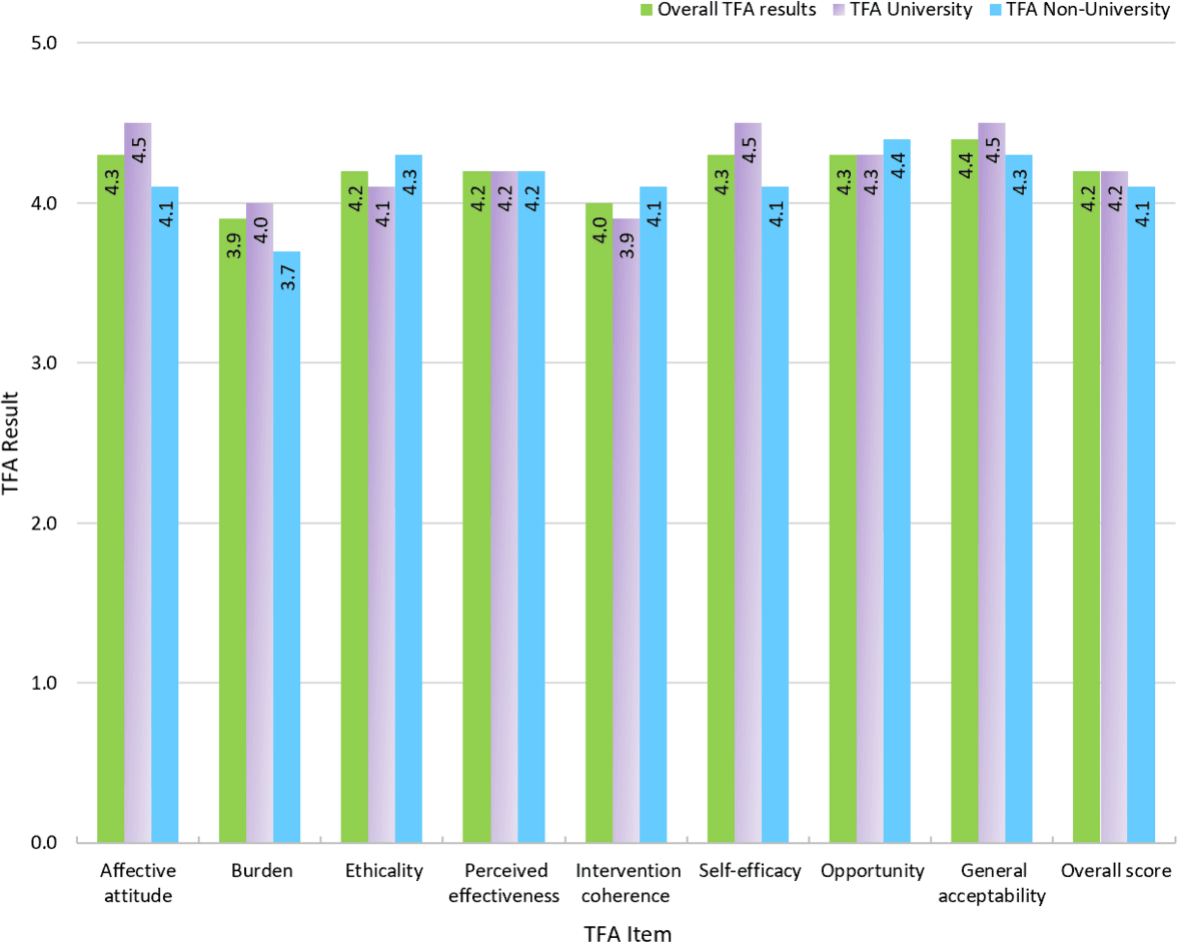
Preoperative TFA results (all items) for the overall population and by education level. TFA: theoretical framework for acceptability.

**Figure 3.**
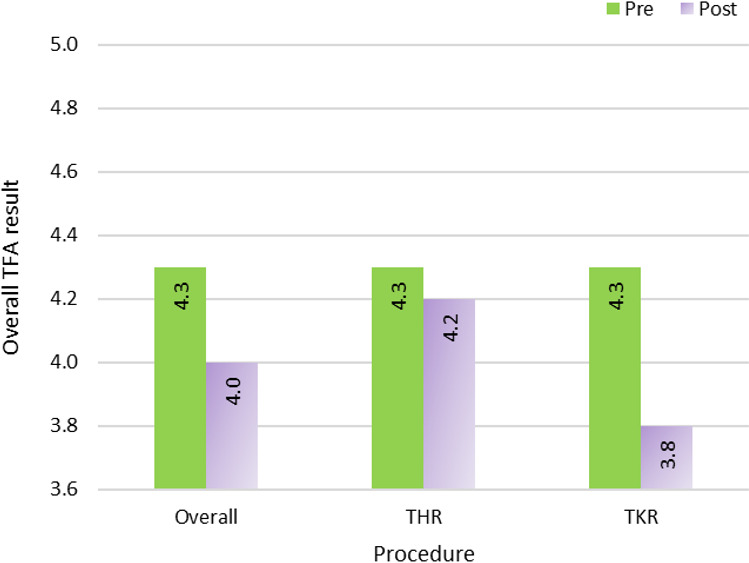
Comparison between pre- and postoperative overall TFA results for the overall population and by procedure. TFA: theoretical framework for acceptability; THR: total hip replacement; TKR: total knee replacement.

The overall score for the preoperative TFA questionnaires was 4.2 (SD 0.6; Table 2). We found a statistically significant increase in self-efficacy with a university level of education compared to non-university (*P*=.04), but no difference related to age, sex, or employment (Figure 2). There was a statistically significant decrease between pre- and postoperative overall TFA (*P*=.007), as well as in pre- and postoperative perceived effectiveness (*P*=.01) and self-efficacy (*P*=.008; Table 3). Furthermore, when looking at TFA results by procedure, there was a statistically significant decrease with TKA in multiple TFA items, including overall acceptability (*P*=.008; Figure 3). There were no statistically significant differences between pre- and postoperative overall TFA nor in any of the TFA items with THA.

**Table 2.** Preoperative TFA^a^ results and subgroup analysis by demographic category^b^.

TFA items	Overall (n=63), mean (SD)	Age			Sex			Education			Employment		
		<70 (n=34), mean (SD)	≥70 (n=29), mean (SD)	*P* value (*t* test)	Male (n=33), mean (SD)	Female (n=30), mean (SD)	*P* value (*t* test)	Nonuniversity (n=28), mean (SD)	University (n=32), mean (SD)	*P* value (*t* test)	Not working (n=36), mean (SD)	Working (n=23), mean (SD)	*P* value (*t* test)
Affective attitude	4.3 (0.9)	4.3 (0.8)	4.4 (0.8)	.84	4.4 (0.8)	4.1 (1.0)	.21	4.1 (1.0)	4.5 (0.8)	.08	4.4 (0.8)	4.3 (1.1)	.67
Burden	3.9 (0.9)	3.9 (1.0)	3.8 (0.9)	.63	4.0 (0.7)	3.9 (1.1)	.47	3.7 (1.2)	4.0 (0.7)	.20	3.8 (1.0)	4.0 (0.9)	.63
Ethicality	4.2 (1.0)	4.1 (1.1)	4.3 (1.1)	.32	4.1 (1.1)	4.3 (0.8)	.34	4.3 (0.9)	4.1 (1.1)	.54	4.1 (1.1)	4.4 (0.8)	.22
Perceived effectiveness	4.2 (0.8)	4.1 (1.0)	4.3 (0.6)	.23	4.2 (0.9)	4.2 (0.9)	.96	4.2 (0.8)	4.2 (0.9)	.98	4.2 (0.7)	4.3 (1.2)	.55
Intervention coherence	4.0 (0.9)	3.8 (1.1)	4.3 (0.6)	.05	4.0 (0.9)	4.0 (1.0)	.99	4.1 (0.7)	3.9 (1.1)	.49	4.1 (0.8)	3.8 (1.2)	.21
Self-efficacy	4.3 (0.7)	4.3 (0.9)	4.3 (0.5)	.93	4.3 (0.8)	4.3 (0.7)	.99	*4.1 (0.9)*	*4.5 (0.6)*	*.04*	4.3 (0.5)	4.3 (1.1)	.89
Opportunity	4.3 (0.9)	4.2 (1.0)	4.5 (0.7)	.16	4.3 (0.9)	4.3 (0.8)	.88	4.4 (0.7)	4.3 (0.9)	.40	4.4 (0.7)	4.2 (1.0)	.27
General acceptability	4.4 (0.5)	4.3 (0.6)	4.4 (0.5)	.74	4.3 (0.5)	4.4 (0.6)	.87	4.3 (0.5)	4.5 (0.5)	.09	4.3 (0.5)	4.5 (0.6)	.40
Overall score^c^	4.2 (0.6)	4.1 (0.6)	4.3 (0.5)	.22	4.2 (0.5)	4.2 (0.7)	.78	4.1 (0.6)	4.2 (0.5)	.55	4.2 (0.5)	4.2 (0.6)	.94

a TFA: theoretical framework for acceptability.

b Statistically significant results are given in italic.

c Burden, ethicality, and opportunity costs were reversed to compute the overall TFA score.

**Table 3.** Change in TFA^a^ from preoperative to 3 months post operation^b^.

TFA items	Overall (n=49)			TKA^c^ (n=27)			THA^d^ (n=22)		
	Pre, mean (SD)	Post, mean (SD)	*P* value (*t* test)	Pre, mean (SD)	Post, mean (SD)	*P* value (*t* test)	Pre, mean (SD)	Post, mean (SD)	*P* value (*t* test)
Affective attitude	4.5 (0.6)	4.2 (1.3)	.09	4.5 (0.6)	4.1 (1.3)	.22	4.6 (0.7)	4.3 (1.3)	.26
Burden	4.0 (0.8)	4.0 (1.1)	.91	3.9 (1.0)	3.8 (1.3)	.70	4.1 (0.7)	4.3 (0.8)	.36
Ethicality	4.3 (0.9)	3.9 (1.3)	.11	*4.4 (0.9)*	*3.6 (1.4)*	*.04*	4.2 (0.9)	4.3 (1.1)	.89
Perceived effectiveness	*4.3 (0.8)*	*3.8 (1.3)*	*.01*	*4.3 (0.7)*	*3.7 (1.4)*	*.02*	4.2 (0.9)	4.0 (1.2)	.28
Intervention coherence	4.1 (0.9)	3.8 (1.2)	.09	*4.2 (0.8)*	*3.6 (1.3)*	*.01*	3.9 (1.1)	4.0 (1.0)	.69
Self-efficacy	*4.4 (0.5)*	*4.0 (1.2)*	*.008*	*4.4 (0.5)*	*3.8 (1.4)*	*.02*	4.5 (0.6)	4.3 (0.9)	.21
Opportunity	4.4 (0.7)	4.1 (1.1)	.08	4.4 (0.7)	4.0 (1.3)	.10	4.4 (0.8)	4.3 (0.7)	.54
General acceptability	4.4 (0.5)	4.3 (1.0)	.56	4.4 (0.5)	4.1 (1.1)	.28	4.4 (0.5)	4.5 (0.8)	.63
Overall score^e^	*4.3 (0.5)*	*4.0 (0.8)*	*.007*	*4.3 (0.5)*	*3.8 (0.9)*	*.008*	4.3 (0.4)	4.2 (0.6)	.41

a TFA: theoretical framework for acceptability.

b Statistically significant results are given in italic.

c TKA: total knee arthroplasty.

d THA: total hip arthroplasty.

e Burden, ethicality, and opportunity costs were reversed to compute the overall TFA score.

### Satisfaction

**Figure 4.**
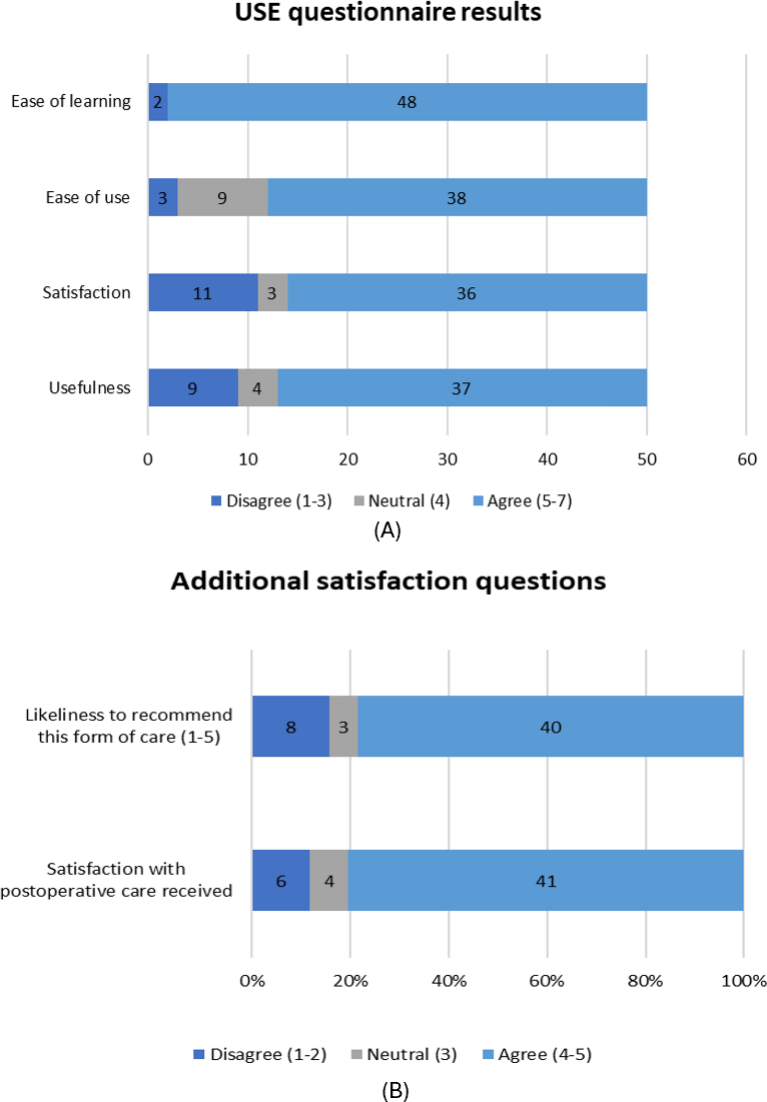
Satisfaction questionnaire results: (A) USE questionnaire results on a stacked bar chart for Likert data; (B) additional satisfaction question results on a 100% stacked bar chart. USE: Usefulness, Satisfaction, and Ease of Use.

The USE questionnaire and the additional satisfaction questions revealed good levels of satisfaction with the use of the app (Table 4 and Figure 4). Patients consistently rated the app as useful and easy to integrate into their recovery process.

**Table 4.** Usefulness, Satisfaction, and Ease of Use questionnaire results 3 months post operation.

Variable	Participants (n=51)
Usefulness score (1-7)	
Disagree (1-3), n (%)	9 (18)
Neutral (4), n (%)	4 (8)
Agree (5-7), n (%)	37 (74)
Missing, n (%)	1 (1.9)
Mean (SD)	5.0 (1.5)
Satisfaction (1-7)	
Disagree (1-3), n (%)	11 (22)
Neutral (4), n (%)	3 (6)
Agree (5-7), n (%)	36 (72)
Missing, n (%)	1 (1.9)
Mean (SD)	4.9 (1.5)
Ease of use (1-7)	
Disagree (1-3), n (%)	3 (6)
Neutral (4), n (%)	9 (18)
Agree (5-7), n (%)	38 (76)
Missing, n (%)	1 (1.9)
Mean (SD)	5.4 (1.1)
Ease of learning (1-7)	
Disagree (1-3), n (%)	2 (4)
Neutral (4), n (%)	0 (0)
Agree (5-7), n (%)	48 (96)
Missing, n (%)	1 (1.9)
Mean (SD)	5.9 (0.9)
Additional questions	
Level of satisfaction with postoperative care (1-5)	
Disagree (1-2), n (%)	6 (12)
Neutral (3), n (%)	4 (8)
Agree (4-5), n (%)	41 (80)
Mean (SD)	4.1 (1.2)
Likelihood to recommend care to friend or family member (1-5)	
Disagree (1-2), n (%)	8 (16)
Neutral (3), n (%)	3 (6)
Agree (4-5), n (%)	40 (78)
Mean (SD)	4.2 (1.3)

### Patient-Reported Outcome Measures

**Figure 5.**
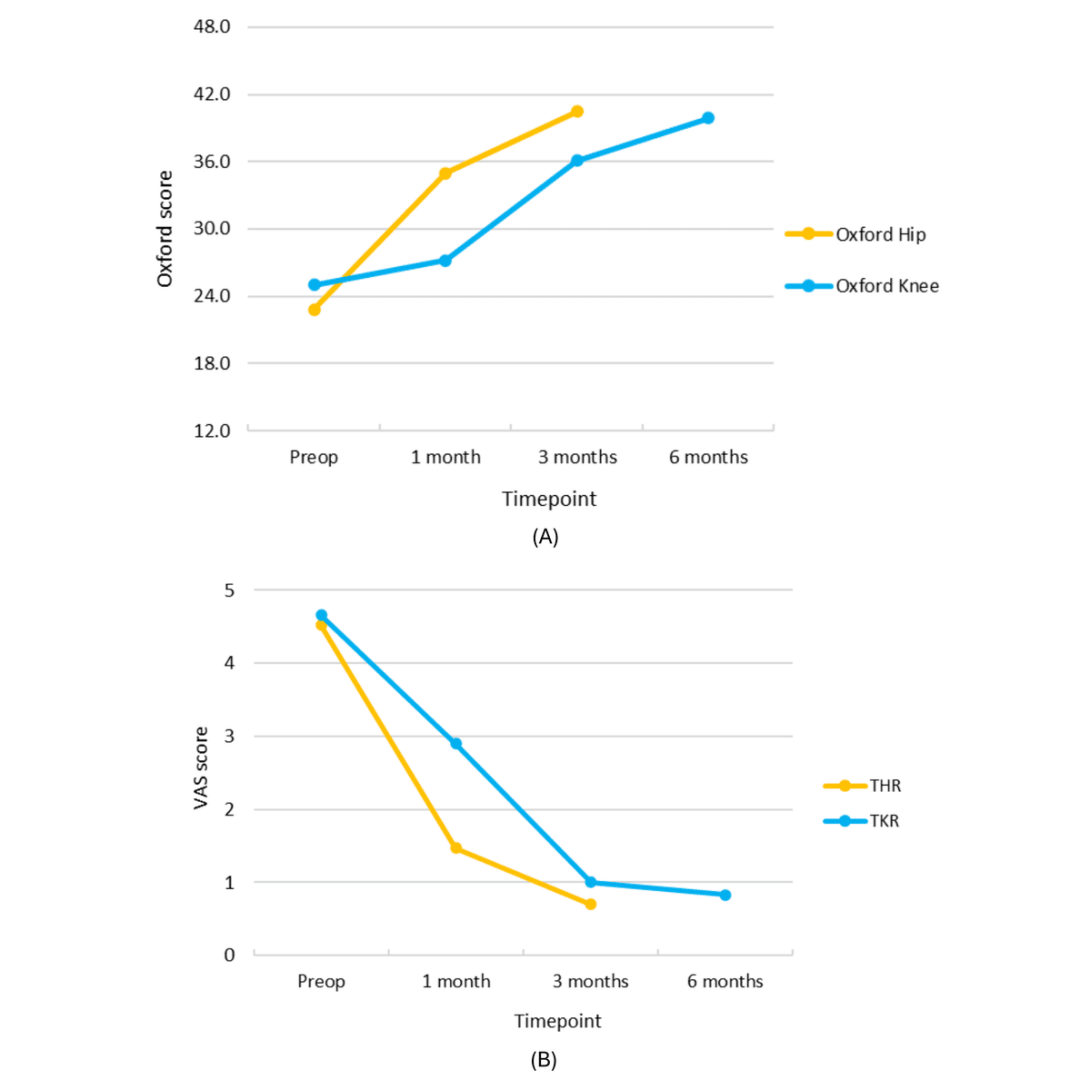
Pre- and postoperative (1, 3, and 6 mo) patient-reported outcome measure results by procedure: (A) Oxford scores and (B) VAS scores. THR: total hip replacement; TKR: total knee replacement; VAS: visual analog scale.

There was a statistically significant increase in overall Oxford score and OHS at 31.2 days post operation, but only at 89.4 days post operation in OKS (Table 5 and Figure 5). As for the VAS, there was a significant decrease in overall, hip, and knee VAS scores at 31.2 days post operation.

**Table 5.** Oxford scores and VAS^a^ results at 1, 3, and 6 months post operation^b^.

Sample time point	Preop^c^		First postop (with preop data)^d^				Second postop (with preop data)^e^				Third postop (with preop data)^f^			
	Participants, n	Mean (SD)	Participants, n	Mean (SD)	Mean difference vs pre	*P* value (*t* test)	Participants, n	Mean (SD)	Mean difference vs pre	*P* value (*t* test)	Participants, n	Mean (SD)	Mean difference vs pre	*P* value (*t* test)
Oxford score overall (0‐48)	49	24.1 (10.1)	49	*30.4 (8.6)*	*6.3*	*<.001*	21	*37.8 (5.7)*	*10.4*	*<.001*	9	*39.1 (3.8)*	*12.2*	*.002*
Oxford Hip Score (0‐48)	20	22.8 (11.8)	20	*35.0 (6.4)*	*12.3*	*<.001*	8	*40.5 (4.4)*	*13.1*	*.01*	1	—^g^	—	—
Oxford Knee Score (0‐48)	29	25.0 (8.9)	29	27.2 (8.6)	2.3	.17	13	*36.1 (5.9)*	*8.7*	*.001*	8	*39.9 (3.3)*	*12.5*	*.004*
VAS overall (0‐10)	49	4.6 (2.6)	49	*2.3 (2.1)*	*−2.3*	*<.001*	22	*0.9 (1.1)*	*−3.1*	*<.001*	9	*1.1 (0.4)*	*−2.4*	*.007*
VAS hip (0‐10)	20	4.5 (2.6)	20	*1.5 (1.4)*	*−3.0*	*<.001*	9	*0.7 (0.3)*	*−2.7*	*.009*	1	—	—	—
VAS knee (0‐10)	29	4.7 (2.6)	29	*2.9 (2.2)*	*−1.8*	*<.001*	13	*1.0 (0.4)*	*−3.4*	*<.001*	8	*0.8 (0.3)*	*−2.7*	*.004*

a VAS: visual analog scale.

b Statistically significant results are given in italic.

c Gap with surgery (days): mean 18.8 (SD 10.1, range 3-36).

d Gap with surgery (days): mean 31.2 (SD 2.8, range 30-44).

e Gap with surgery (days): mean 89.4 (SD 5.1, range 67-93).

f Gap with surgery (days): mean 180.1 (SD 0.4).

g Not available.

### App Nondownload Rate

Of the 25 participants who did not download the app, 7 provided a reason. Their responses included technological concerns (n=2), feeling preoccupied and anxious preparing for the procedure (n=1), family issues (n=2), missing download instructions (n=1), and privacy concerns (n=1). The remaining 18 participants were also contacted on multiple occasions but never responded.

## Discussion

### Principal Findings

In this study, we investigated the acceptability of the use of the mymobility app for the postoperative care of 100 participants undergoing THA and TKA. We sought to investigate acceptability using the TFA to ensure all aspects of acceptability were measured.

Preoperatively, overall acceptability was satisfactory, with all but one TFA item scoring above 4 out of 5. The sole exception was burden (3.9/5), which may reflect lower familiarity with smartphone apps among older participants in our sample. We also found that higher education was associated with a higher level of self-efficacy. While general and overall acceptability were not significantly higher with higher education, this trend aligns with Wang et al’s [15], Nuveo et al’s [31], and Lee et al’s [32] results, who described trends of higher engagement and adherence with higher education and lower receptivity to digital health technologies with lower education. Thus, these findings reveal the importance for app developers to consider this gap and tailor their content to diverse populations, notably through the inclusion of images or videos, which are more easily accessible than heavy-text content.

When comparing pre- and postoperative TFAs, although overall scores were satisfactory at both time points, we found a significant decrease in overall acceptability postoperatively. We suspect this decrease is attributed to elevated expectations toward the app, which were adjusted following its use. However, we believe acceptability could also have been influenced by the natural process of recovery. Indeed, the decline in TFA was significant in TKA, but not in THA. PROM results showed the same trend, with no improvement until 3 months post operation in TKA as opposed to 1 month post operation in THA. This is consistent with Booth et al’s [19] and Bourne et al’s [33] results showing that TKA patients felt a higher need for in-person rehabilitation as opposed to THA and that short-term outcomes are superior in THA than in TKA. When considering the TFA items that decreased in TKA, which are linked to the perceived helpfulness of the app, this pushes us to believe that the decrease in postoperative acceptability was likely also driven by the surgical outcomes. This illustrates the possibility for app developers to better address the challenges in TKA rehabilitation, notably through adjustment of patient expectations following this procedure.

When looking at participants who did not download the app, reasons included concerns regarding technology in 2 cases, which might be due to the age of the participants (77-79 y). Additionally, 1 participant expressed privacy concerns. Although we explained how data collection and privacy functioned within the app, we still expected this number to be higher. This result is a positive sign for patient trust toward platforms used to provide care, although the need to discuss patient confidentiality within the app remains important.

### Comparison With Previous Work

Multiple studies have investigated patient perspectives on the use of mHealth apps for joint arthroplasty. These studies all reported a high level of engagement, adherence, and satisfaction [13,15-19,31]. Factors that influenced the results positively included higher education and supportive environments, whereas those that did negatively included higher age, lower economic status, and worsened physical or psychological condition [15,17,31]. Patients also described better health care accessibility and reduced isolation with these interventions [20]. Our results showed that higher education was associated with greater acceptability. However, unlike prior work, we did not observe other demographic characteristics to significantly influence acceptability.

### Strengths and Limitations

The main strength of this study is the measure of acceptability using a validated tool (TFA), which allowed us to assess all domains of this concept. Using this framework, we measured acceptability preoperatively, prior to exposure to the app, and postoperatively, after using the app. This allowed us to interpret how preexposure expectations were met following use of the app. Other strengths include the use of a single app, which reduced heterogeneity; the ITT design, which reflects real-world adherence; and the diverse population.

This study also has limitations. The main limitation is the absence of a control group, which limits the ability to attribute the results specifically to the app. This limitation is a result of ethical concerns brought by our institutional review board, which prohibited us from having a control group that could be denied a beneficial intervention. We also observed a high rate of unfilled questionnaires, which could have created a response bias. We believe this is due to most questionnaires being distributed by email or phone, creating a limiting step in communication. Other important limitations include the use of a single app, limiting the generalizability of the results; a potential selection bias toward patients willing to use the app; and statistical analysis being only performed with *t* tests, which might have limited the power of the conclusions. In addition, there was no assessment of baseline technological literacy, which could have affected results despite the app’s technological guidance features.

### Conclusion

There was a good level of acceptability with the use of mymobility for the postoperative management of THA and TKA, although acceptability decreased postoperatively. This decrease could signify high expectations toward the app preoperatively or higher than expected difficulty and pain in the early postoperative period. Higher education was associated with higher preoperative acceptability. TKA was associated with lower postoperative acceptability, which could be related to the delayed recovery in TKA when compared to THA. These 2 trends have previously been described in the literature, indicating potential gaps to address for app developers. We found a good level of satisfaction with the app, and Oxford scores showed better outcomes in THA than TKA, whereas VAS showed equally significant decreases in pain with both procedures. Privacy concerns were rare, with only 1 patient reporting them.

The impact of the natural recovery process in total joint arthroplasty on the acceptability of mHealth remains unclear. This gap could be addressed through a comparison of acceptability pre- and postoperatively in cohorts using the app and cohorts with standard care.

## Supplementary material

10.2196/79682 Multimedia Appendix 1 Overview of data collected by the research team and by the mymobility app.

10.2196/79682 Multimedia Appendix 2 Overview of the mymobility functions and interface. (A) Features of the app with depictions of the app interface and (B) Walk-AI, the mymobility artificial intelligence–assisted gait speed analysis predicting and determining level of recovery based on gait speed.

10.2196/79682 Multimedia Appendix 3 Modified theoretical framework for acceptability questionnaire.

10.2196/79682 Multimedia Appendix 4 Usefulness, Satisfaction, and Ease of Use questionnaire.

10.2196/79682 Multimedia Appendix 5 Additional satisfaction questions.

10.2196/79682 Multimedia Appendix 6 Oxford Hip and Oxford Knee Scores.

10.2196/79682 Multimedia Appendix 7 Visual analog scale for pain.

## Abbreviations

mHealth: mobile health
OHS: Oxford Hip Score
OKS: Oxford Knee Score
PROM: patient-reported outcome measure
TFA: theoretical framework for acceptability
THA: total hip arthroplasty
TKA: total knee arthroplasty
USE: Usefulness, Satisfaction, and Ease of Use
VAS: visual analog scale
