# Correction: Acceptability of a Digital Care App in Patients Undergoing Hip and Knee Arthroplasty: Prospective Cohort Study

**DOI:** 10.2196/101097

**Published:** 2026-08-14

**Authors:** Yacine Louni, Matthew Laroche, Abdulrhman Alnasser, Mohammad Abuhaneya, Eric Belzile, Sandhya Baskaran, Jennifer Mutch, Anthony Albers

**Affiliations:** 1 Faculty of Medicine, McGill University, Montreal, QC, Canada; 2 Division of Orthopaedic Surgery, McGill University, St Mary’s Hospital, 3830 Avenue Lacombe, Montreal, QC, H3T 0A2, Canada, +1 514-345-3511 ext 3282; 3 St Mary's Hospital Research Center, St Mary's Hospital, Montreal, QC, Canada

In “Acceptability of a Digital Care App in Patients Undergoing Hip and Knee Arthroplasty: Prospective Cohort Study” [1], the authors noted one error.

The credentials of author Sandhya Baskaran originally appeared as:

*MSc*

These are now corrected to:

*MEd, MBA*

The correction will appear in the online version of the paper on the JMIR Publications website, together with the publication of this correction notice. Because this was made after submission to PubMed, PubMed Central, and other full-text repositories, the corrected article has also been resubmitted to those repositories.
