# Supplementary material for: Acceptability of a Digital Care App in Patients Undergoing Hip and Knee Arthroplasty: Prospective Cohort Study
Source: JMIR Hum Factors. 2026 Jan 27;13:e79682. doi: 10.2196/79682 (PMC12844828; doi:10.2196/79682)
Supplement: Multimedia Appendix 1 [file humanfactors-v13-e79682-s001.docx]

| Data Category | Research team | Mymobility® application |
| --- | --- | --- |
| Personal data | - Name - Age - Sex assigned at birth - Ethnicity - Education level - Employment - Area of residency (Urban/Rural) | - Name - Address - Email address - Payment information Picture - Voice recording - Telephone number |
| Treatment data | - Type of surgery undergone (THA vs TKA) - Previous surgeries or injuries - Previous physical examination by surgeon | - Treatment information - App usage - Mobility Gait quality - Heart rate |
