# Supplementary figures and images for: Acceptability of a Digital Care App in Patients Undergoing Hip and Knee Arthroplasty: Prospective Cohort Study
Source: JMIR Hum Factors. 2026 Jan 27;13:e79682. doi: 10.2196/79682 (PMC12844828; doi:10.2196/79682)

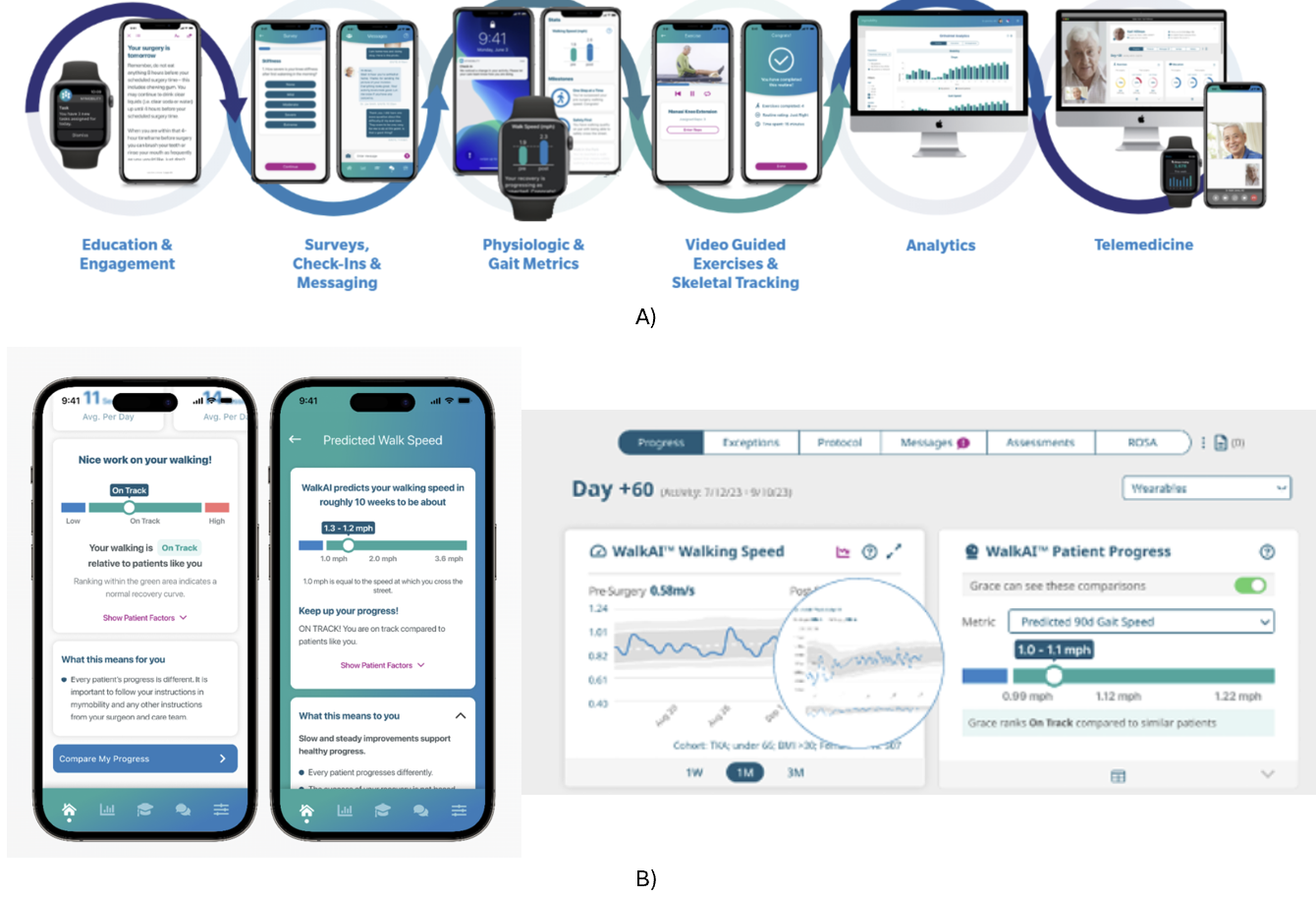

Supplement: Multimedia Appendix 2 [file humanfactors-v13-e79682-s002.docx]

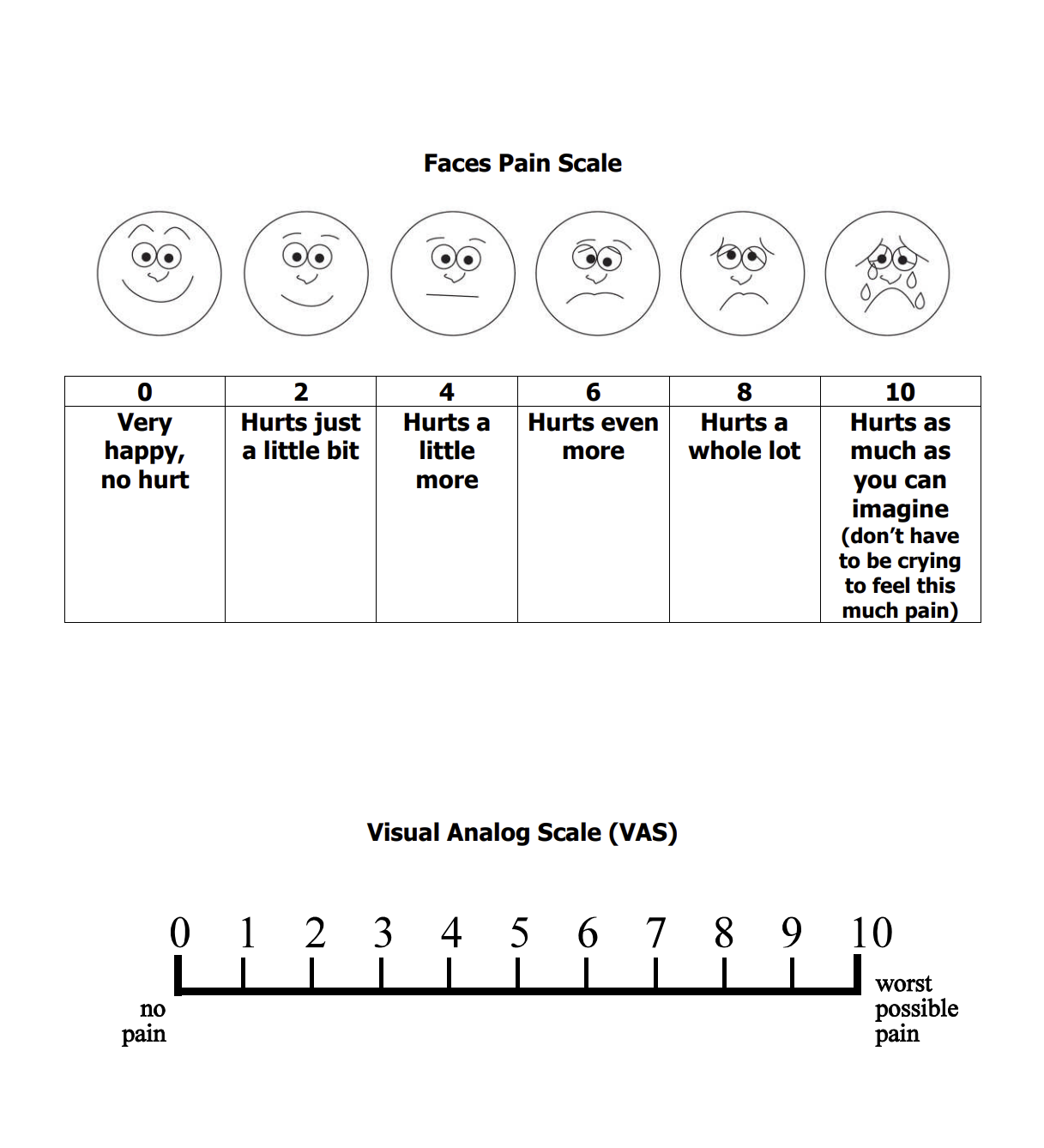

Supplement: Multimedia Appendix 7 [file humanfactors-v13-e79682-s007.doc]
