# Supplementary material for: Acceptability of a Digital Care App in Patients Undergoing Hip and Knee Arthroplasty: Prospective Cohort Study
Source: JMIR Hum Factors. 2026 Jan 27;13:e79682. doi: 10.2196/79682 (PMC12844828; doi:10.2196/79682)
Supplement: Multimedia Appendix 3 [file humanfactors-v13-e79682-s003.doc]

| TFA construct | TFA questionnaire items |
| --- | --- |
| **Affective attitude**  *How an individual feels about the intervention* | a. Prospectively: How comfortable do you feel to use the mymobility application for your post operative care?  b. Retrospectively: How comfortable did you feel to use the mymobility application for your post operative care?   | Very uncomfortable | Uncomfortable | No opinion | Comfortable | Very comfortable | | --- | --- | --- | --- | --- | | 1 | **2** | **3** | **4** | **5** | |
| **Burden (This item was inverted in the final score)**  *The amount of effort required to participate in the intervention* | a. Prospectively: How much effort do you think it will take to engage with the myMobility application for your post operative care?  b. Retrospectively: How much effort did it take to engage with the myMobility application for your post operative care?   | No effort at all | A little effort | No opinion | A lot of effort | Huge effort | | --- | --- | --- | --- | --- | | 1 | **2** | **3** | **4** | **5** | |
| **Ethicality (This item was inverted in the final score)**  *The extent to which the intervention has good fit with an individual’s value system* | a. Prospectively: There are moral or ethical consequences in using the myMobility application for post operative care.  b. Retrospectively: There are moral or ethical consequences in using the myMobility application for post operative care.   | Strongly disagree | Disagree | No opinion | Agree | Strongly agree | | --- | --- | --- | --- | --- | | 1 | **2** | **3** | **4** | **5** | |
| **Perceived effectiveness**  *The extent to which the intervention is perceived to have achieved its objective* | a. Prospectively: The use of the myMobility application will be a helpful tool in my recovery following my surgery.  b. Retrospectively: The use of the myMobility application has been a helpful tool in my recovery following my surgery.   | Strongly disagree | Disagree | No opinion | Agree | Strongly agree | | --- | --- | --- | --- | --- | | 1 | **2** | **3** | **4** | **5** | |
| **Intervention coherence**  *The extent to which the participant understands how the intervention works* | a. Prospectively: It is clear to me how the use of myMobility will help in my recovery after my surgery.  b. Retrospectively: It is clear to me how the use of myMobility has helped in my recovery after my surgery   | Strongly disagree | Disagree | No opinion | Agree | Strongly agree | | --- | --- | --- | --- | --- | | 1 | **2** | **3** | **4** | **5** | |
| **Self-efficacy**  *A participant’s confidence that they can perform behaviour(s) required to participate in the intervention* | a. Prospectively: How confident do you feel about being able to use myMobility for your process of recovery following your surgery.  b. Retrospectively: How confident dis you feel about being able to use myMobility for your process of recovery following your surgery.   | Very unconfident | Unconfident | No opinion | Confident | Very confident | | --- | --- | --- | --- | --- | | 1 | **2** | **3** | **4** | **5** | |
| **Opportunity costs (This item was inverted in the final score)**  *The benefits, profits or values that would have to be given up to engage with the intervention* | a. Prospectively: Using myMobility will interfere with my other priorities.  b. Retrospectively: Using myMobility interfered with my other priorities.   | Strongly disagree | Disagree | No opinion | Agree | Strongly agree | | --- | --- | --- | --- | --- | | 1 | **2** | **3** | **4** | **5** | |
| **General acceptability** | a. Prospectively: How acceptable is the use of myMobility for post operative care to you?  b. Retrospectively: How acceptable was the use of myMobility for post operative care to you?   | Completely unacceptable | Unacceptable | No opinion | Acceptable | Completely acceptable | | --- | --- | --- | --- | --- | | 1 | **2** | **3** | **4** | **5** | |
