# Supplementary material for: Acceptability of a Digital Care App in Patients Undergoing Hip and Knee Arthroplasty: Prospective Cohort Study
Source: JMIR Hum Factors. 2026 Jan 27;13:e79682. doi: 10.2196/79682 (PMC12844828; doi:10.2196/79682)
Supplement: Multimedia Appendix 4 [file humanfactors-v13-e79682-s004.doc]

All the questions for this questionnaire were graded using a 7-point Likert scale:

| Strongly disagree | Disagree | Disagree | No opinion | Agree | Agree | Strongly agree |
| --- | --- | --- | --- | --- | --- | --- |
| 1 | **2** | **3** | **4** | **5** | **6** | **7** |

| USE categories | USE questionnaire items |
| --- | --- |
| **Usefulness** | • It helps me be more effective.  • It helps me be more productive.  • It is useful.  • It gives me more control over the activities in my life.  • It makes the things I want to accomplish easier to get done.  • It saves me time when I use it.  • It meets my needs.  • It does everything I would expect it to do. |
| **Satisfaction** | • I am satisfied with it.  • I would recommend it to a friend.  • It is fun to use.  • It works the way I want it to work.  • It is wonderful.  • I feel I need to have it.  • It is pleasant to use. |
| **Ease of Use** | • It is easy to use.  • It is simple to use.  • It is user friendly.  • It requires the fewest steps possible to accomplish what I want to do with it.  • It is flexible.  • Using it is effortless.  • I can use it without written instructions.  • I don't notice any inconsistencies as I use it.  • Both occasional and regular users would like it.  • I can recover from mistakes quickly and easily.  • I can use it successfully every time. |
| **Ease of Learning** | • I learned to use it quickly.  • I easily remember how to use it.  • It is easy to learn to use it.  • I quickly became skillful with it. |
