# Supplementary material for: Acceptability of a Digital Care App in Patients Undergoing Hip and Knee Arthroplasty: Prospective Cohort Study
Source: JMIR Hum Factors. 2026 Jan 27;13:e79682. doi: 10.2196/79682 (PMC12844828; doi:10.2196/79682)
Supplement: Multimedia Appendix 5 [file humanfactors-v13-e79682-s005.doc]

| 1. How satisfied were you with the postoperative care received?   | Very dissatisfied | Somewhat dissatisfied | Neither satisfied nor dissatisfied | Agree | Strongly agree | | --- | --- | --- | --- | --- | | 1 | **2** | **3** | **4** | **5** | |
| --- | --- | --- | --- | --- | --- | --- | --- | --- | --- | --- |
| 2. How likely will you be to recommend this form of care to a friend or a family member   | Very unlikely | Somewhat unlikely | Neutral | Somewhat likely | Very likely | | --- | --- | --- | --- | --- | | 1 | **2** | **3** | **4** | **5** | |
