# Supplementary material for: Acceptability of a Digital Care App in Patients Undergoing Hip and Knee Arthroplasty: Prospective Cohort Study
Source: JMIR Hum Factors. 2026 Jan 27;13:e79682. doi: 10.2196/79682 (PMC12844828; doi:10.2196/79682)
Supplement: Multimedia Appendix 6 [file humanfactors-v13-e79682-s006.doc]

**All the questions are answer considering the following initial prompt: During the past 4 weeks…**

| Oxford Hip Item | Oxford Knee Item |
| --- | --- |
| 1. How would you describe the pain you usually had from your hip?   | None | Very mild | Mild | Moderate | Severe | | --- | --- | --- | --- | --- | | 0 | **1** | **2** | **3** | **4** | | 1. How would you describe the pain you usually had from your knee?   | None | Very mild | Mild | Moderate | Severe | | --- | --- | --- | --- | --- | | 0 | **1** | **2** | **3** | **4** | |
| 2. Have you had any trouble with washing and drying yourself (all over) because of your hip?   | No trouble at all | Very little trouble | Moderate trouble | Extreme difficulty | Impossible to do | | --- | --- | --- | --- | --- | | 0 | **1** | **2** | **3** | **4** | | 2. Have you had any trouble with washing and drying yourself (all over) because of your knee?   | No trouble at all | Very little trouble | Moderate trouble | Extreme difficulty | Impossible to do | | --- | --- | --- | --- | --- | | 0 | **1** | **2** | **3** | **4** | |
| 3. Have you had any trouble getting in and out of a car or using public transport because of your hip? (whichever you tend to use)   | No trouble at all | Very little trouble | Moderate trouble | Extreme difficulty | Impossible to do | | --- | --- | --- | --- | --- | | 0 | **1** | **2** | **3** | **4** | | 3. Have you had any trouble getting in and out of a car or using public transport because of your knee? (whichever you tend to use)   | No trouble at all | Very little trouble | Moderate trouble | Extreme difficulty | Impossible to do | | --- | --- | --- | --- | --- | | 0 | **1** | **2** | **3** | **4** | |
| 4. Have you been able to put on a pair of socks, stockings or tights?   | Yes, easily | With little difficulty | With moderate difficulty | With extreme difficulty | No, impossible | | --- | --- | --- | --- | --- | | 0 | **1** | **2** | **3** | **4** | | 4. For how long have you been able to walk before pain from your knee becomes severe? (with or without a stick)   | No pain/more than 30 minutes | 16 to 30 minutes | 5 to 15 minutes | Around the house only | Not at all-pain severe on walking | | --- | --- | --- | --- | --- | | 0 | **1** | **2** | **3** | **4** | |
| 5. Could you do the household shopping on your own?   | Yes, easily | With little difficulty | With moderate difficulty | With extreme difficulty | No, impossible | | --- | --- | --- | --- | --- | | 0 | **1** | **2** | **3** | **4** | | 5. After a meal (sat at a table), how painful has it been for you to stand up from a chair because of your knee?   | Not at all painful | Slightly painful | Moderately painful | Very painful | Unbearable | | --- | --- | --- | --- | --- | | 0 | **1** | **2** | **3** | **4** | |
| 6. For how long have you been able to walk before pain from your hip becomes severe? (with or without a stick)   | No pain/more than 30 minutes | 16 to 30 minutes | 5 to 15 minutes | Around the house only | Not at all-pain severe on walking | | --- | --- | --- | --- | --- | | 0 | **1** | **2** | **3** | **4** | | 6. Have you been limping when walking, because of your knee?   | Rarely/never | Sometimes, or just at first | Often, not just at first | Most of the time | All the time | | --- | --- | --- | --- | --- | | 0 | **1** | **2** | **3** | **4** | |
| 7. Have you been able to climb a flight of stairs?   | Yes, easily | With little difficulty | With moderate difficulty | With extreme difficulty | No, impossible | | --- | --- | --- | --- | --- | | 0 | **1** | **2** | **3** | **4** | | 7. Could you kneel down and get up again afterwards?   | Yes, easily | With little difficulty | With moderate difficulty | With extreme difficulty | No, impossible | | --- | --- | --- | --- | --- | | 0 | **1** | **2** | **3** | **4** | |
| 8. After a meal (sat at a table), how painful has it been for you to stand up from a chair because of your hip?   | Not at all painful | Slightly painful | Moderately painful | Very painful | Unbearable | | --- | --- | --- | --- | --- | | 0 | **1** | **2** | **3** | **4** | | 8. Have you been troubled by pain from your knee in bed at night?   | No nights | Only 1 or 2 nights | Some nights | Most nights | Every night | | --- | --- | --- | --- | --- | | 0 | **1** | **2** | **3** | **4** | |
| 9. Have you been limping when walking, because of your hip?   | Rarely/never | Sometimes, or just at first | Often, not just at first | Most of the time | All the time | | --- | --- | --- | --- | --- | | 0 | **1** | **2** | **3** | **4** | | 9. How much has pain from your knee interfered with your usual work (including housework)?   | Not at all | A little bit | Moderately | Greatly | Totally | | --- | --- | --- | --- | --- | | 0 | **1** | **2** | **3** | **4** | |
| 10. Have you had any sudden, severe pain - 'shooting', 'stabbing' or 'spasms' - from the affected hip?   | No days | Only 1 or 2 days | Some days | Most days | Every day | | --- | --- | --- | --- | --- | | 0 | **1** | **2** | **3** | **4** | | 10. Have you felt that your knee might suddenly 'give way' or let you down?   | Rarely/never | Sometimes, or just at first | Often, not just at first | Most of the time | All the time | | --- | --- | --- | --- | --- | | 0 | **1** | **2** | **3** | **4** | |
| 11. How much has pain from your hip interfered with your usual work (including housework)?   | Not at all | A little bit | Moderately | Greatly | Totally | | --- | --- | --- | --- | --- | | 0 | **1** | **2** | **3** | **4** | | 11. Could you do the household shopping on your own?   | Yes, easily | With little difficulty | With moderate difficulty | With extreme difficulty | No, impossible | | --- | --- | --- | --- | --- | | 0 | **1** | **2** | **3** | **4** | |
| 12. Have you been troubled by pain from your hip in bed at night?   | No nights | Only 1 or 2 nights | Some nights | Most nights | Every night | | --- | --- | --- | --- | --- | | 0 | **1** | **2** | **3** | **4** | | 12. Could you walk down one flight of stairs?   | Yes, easily | With little difficulty | With moderate difficulty | With extreme difficulty | No, impossible | | --- | --- | --- | --- | --- | | 0 | **1** | **2** | **3** | **4** | |
